# Review of April No. Chicago Medical Journal

**Published:** 1874-05

**Authors:** Z. C. McElroy

**Affiliations:** Zanesville, O.


ARTICLE IV.—*Review—Chicago Medical Journal, April,* 1874. By a Country Contributor. [Z. C. McElroy, M.D., Zanesville, O.]

The opening article, the Valedictory Address of Prof. Miller, contains a very admirable *resume* of the results of modern scientific and philosophic researches and investigations, stated explicitly, and in many parts eloquently. It should have commenced with the first paragraph on page 195. The statement made on page 200, that the doctrine of evolution excludes creation and theism, is only true when limited time is admitted as a factor. But as time, so called, is only the relative position of the moving heavenly bodies to each other, it is not properly a factor in the premises at all. "God pays in full, but not every Saturday night," is a homely way of excluding time from some of our calculations. "The mill of God grinds slow, but fine," is another mode of expressing the same idea.

The Professor has evidently not paused to study patiently, and in all its bearings, in the light of modern science, the quotation he introduces from the Bible, on page 201. Nothing is said about time in it; not likely to be an accidental omission in the inspired text.

The first paragraph on page 198 should have quotation marks at its commencement, and after the interrogation point at the end of the second paragraph, and possibly at other places.

Prof. Miller, like some clergymen, seems not to have known when his address was ended, or at least that part of it extending from pages 193 to 202, relating to the conservation of energy, or he would have omitted all on pages 202 to 205. He had said enough to arouse the attention of his graduating class to apply themselves to the work of ascertaining how far and to what extent the great truths he had just stated would affect practical medicine. There he should have left them, each free to work for himself. Instead of that, however, he points out lines of investigation which must bring them back into the same old ruts from which they started. Better for them it would have been had he told them, in the language of Helmholtz, "*Wer bei der Verfolgung der Wissenschaften nach unmittelbaren praktischen Nutzen jagt, Kann Zeimlich sicher sein dass er vergebens jagen wird.*"*

It is to my mind a great blemish on an otherwise really excellent and thoughtful address, to have made such statements as those in regard to the "chemistry" of "disease," as well as the "poisons" that cause them, and the chemical means of "cure," which appear in it.

Everything pertaining to living beings and things merge themselves into questions of materials, forms of organic structure, and the forces concerned, or modes of force concerned, in their evolution, and decay or loss. "Disease," "poisons," and "cure," are words which appear very properly in "Good's Study of Medicine," but are certainly out of place in casting the horoscope of medicine in the presence of the great general principles he had just annunciated.

On the whole, the address is excellent, and but for these defects, would do credit to any professor in any medical school in existence.

The second article—Myelitis—by Professor Hay, a model of exact and careful observation, and record of results, is introduced to settle a diagnostic point—for the case has already been

* "He who in the pursuit of science seeks directly practical applications, can be pretty sure that he will seek in vain."—*J. Matthews Duncan*, *Address at Edinburgh*, *December*, 1873.

reported under another name—supplying evidence for the ten thousand millionth time, that to map, and fix with exactness, the disturbances of the moving equilibrium on which life depends, has always been a failure in the past, and will continue to fail to the end. Doubtless Prof. Hay is right in his diagnosis so far as he goes, but he falls short of "the whole truth;" to show which, it is only necessary to inquire if the balance of the patient—excluding his brain and spinal cord—was well. His exact observations show conclusively that the disturbed equilibrium of the patient's brain and spinal cord was only an incident in the disturbance of the forms of organic structure, and the equilibrium of motion in his structures generally. Was the "myelitis" a cause, a consequence, or coincidence of the universal disturbance of structure, and motion in structure, as evidenced by the changes of function recorded by him?

The facts probably were, that there were wide-spread modifications and losses of "function," which would not and could not have been the case had the patient's structures had their normal molecular arrangement of material, and motion of material. The mean of the various velocities of motion in his structures, as evidenced by his temperature, was natural, and that was about the single feature the patient presented that was so.

The neighboring "practitioner," incidentally called in, who prescribed very properly for the "disease"—pain—evidently without studying the case, was, doubtless, and unintentionally, instrumental in hastening the end. It occurs to me, however, that in the professional management of the "disease" the patient was partially, if not wholly, lost sight of; affording evidence, again, of the misleading character of the "London College's" map of 900 separate and distinct "diseases," so recently published, and so widely respected. The real state of things existing at the time of Prof. Hay's first visit, or connection with the case, was that structural forms of material were extensively lost, or changed, as evidenced by changed and lost functions, so concisely described by him.

The original or remote cause, in all probability, of this derangement of structure, was a failure on the part of the patient's structures themselves to provide for their own reproduction from new material as they were wasted by function; or a failure of the

"lymphatic system" to collect the material in which living structures store up the so-called "vital force," and restore it, with new material, at the proper time and place, near the right auricle. This is rendered all the more certain by the patient's age, 65, for at that time of life the structures are naturally "failing," and if they did not fail, none of us would ever grow old, or die—we would be immortal.

From Prof. Hay's admirable description of lost and modified functions—behind which there were modifications, or loss of the natural structural arrangement of material—it seems to me that the primary failure was in the lymphatic system. The patient's age, the season of the year, etc., all point in this direction.

The third article, by Dr. Stewart, abounds in evidence of the mistake made when the "disease" is the object of professional management, instead of the patient. It is so easy to play marbles, and so difficult to play chess, especially with a skillful opponent. We take up this marble—some drug or medicine or combination—and "plump" it at the "disease;" if it misses, try another, and so on. Why not? As the boys say, "aint there fun in shooting, whether you hit or miss?" Not so in chess. Like the effective work in nature this is played in secrecy and silence, the results, in the motions of the "men" are only apparent.

In Dr. Stewart's case there was from the first hopelessly lost or modified structural arrangements of the materials of his patient's body. He played marbles with it, and failed to win the game. Had he played chess, with Stricker, and Rindfleisch, and Van der Kolk, as instructors, his patient would have taken less "drugs and medicines," and his professional interference would have been confined to "possibilities," or "probabilities," with less "chance" in the game. Modifications, or loss of the normal structural arrangement of living bodies, upon which function depends, cannot always be restored by "drugs and medicines." All the positive evidence on the subject is in the negative. Symptoms and appearances may sometimes favor such a "belief," but external demonstrations are against it. The "scars" alluded to on page 227 (No. 7) do not favor such a "belief." Amputated limbs are never reproduced. Opacity of the lens in the eye is only remediable by mechanically removing it.

Still, working in the accredited channels, Dr. Stewart treated his patient industriously, if not successfully. I do not censure him. Twenty years since, in accordance with my instructions, I treated "diseases." Now, I regard my patients as altogether of more importance than their "diseases."

The fourth article, by Dr. English, is only interesting as showing just what has happened in the development of a fœtus when some of its "organic forms of structure" were left out, in consequence of deficient or misdirected force.

PROGRESS IN MEDICAL SCIENCES.

The first article, Neuro Pathology and Psychological Medicine, by Prof. Hay, is a statement of the "conclusions" of Dr. Mendel in regard to "differentially" diagnosing typhus from insanity, affording evidence, again, of the utter inutility of efforts to affix exactness to the shifting and ever changing phenomena when the equilibrium of health is disturbed in different bodies, no two of whom ever were or are likely to be "exactly alike." The facts in regard to typhus and insanity are, that in both there is always disturbance of structural arrangements of material, and sometimes loss, as evidenced by changed or lost functions. Dr. Barnes, in his recent work on "The Diseases (?) of Women," says, in regard to "neuralgia" of the uterus, "histeralgia," etc., that they are terms calculated in their use to "lull the spirit of inquiry by fostering the belief that they embody a pathological entity." And so of typhus and insanity, "they took their rise at a period when the precise and minute investigations at present in vogue were comparatively unknown," (Barnes, p. 99,) and therefore "no longer satisfy any but those who are satisfied with the imperfect pathological knowledge of the past."

2. Dr. Eulenberg's case, again, shows not a "disease," but a modification of the structural arrangement of the tissues of a boy by mechanical violence, as evidenced by permanently changed functions.

3. So of Dr. Charcot's observations; all concur in showing changes, or loss of the structural arrangement of material, behind changed or lost functions.

4. Prof. Ferrier's physiological experiments, in their results, point to the same general facts.

5. Dr. Hallock's suggestions in regard to hospitals for the insane are in accordance with the experiences of our late war, that barracks, or hospitals, not for the insane alone, but for all other sickness, is one of the mistakes of the past.

The 2nd article, by Dr. Jackson, offers nothing for special remark, but is, nevertheless, interesting in several particulars, as anomalous statistics, etc., in reference to gynæcology.

The 3rd article, by Lecturer Owens, is of the same general character in reference to surgery. Its feature, however, is the emphatic confession of Prof. Gross of our total ignorance of the pathogenesis of tumors. Prof. Gross' confession is both timely and truthful; and as it takes "thunder" to wake some of us up, is an important addition to the "capital stock" of us agitators for an "advance" in our conceptions of physiological and pathological processes, and the therapeutics which will naturally follow them. Prof. Gross makes an effective iconoclast. Instead of casting my lot with his "wandering or mobile connective-tissue cells," I prefer to study force, as it is "stored" up in material, as the central feature of all tissue, natural or neoplasms, physiological or pathological.

*Hospital Report*, by Surgeon Lyman. Gynæcological reports are, by some of us, naturally regarded with suspicion. As directing attention almost exclusively to deceptive local appearances, they are mischievous; for many of them disappear spontaneously, while some are probably hastened in their departure by local in connection with general treatment. Like the government published report of "Columbia Hospital," they make good "padding." Dr. Barnes, in his just published work on Women, iterates and re-iterates the fact that structural changes are always found behind changed functions. And "structural changes" are not to be rectified by local applications through speculums alone. The Editor-in-chief (Prof. Allen) has told a good story in reference to an elderly practitioner who called upon him for information in regard to a proper location, as he had been compelled to abandon his life-long field of labor by a new doctor who used a —— horn, and had taken all his practice. His advice was significant of his opinion of indiscriminate "speculum practice." Address Prof. Allen for particulars.

*The Clinic*, by Lecturer Bridge, supplies some needed knowledge in regard to the part played by the little trichina insect in changing the structural arrangement of living tissue, demonstrating that changes of function are inseparably associated with changes of structure. The changes effected by the parasite in living tissue, so far as function is concerned, differs in no respect from that by "sclerosis," (so called) except in the possibility of patients pulling out of trichinosis, which never occurs after "sclerosis."

*Society Reports*—That of the Chicago Medical Society has been anticipated, to some extent, by the publication of Dr. Lyman's report on gynæcology.

Prof. Owens' report points out the usual structural changes behind changes of function in his case of "Parietal Gaseous Abscess," which, it seems to me, offered a good opportunity for using the "aspirator."

Dr. Heydock's case of empyema also presented an excellent opportunity for using an "aspirator," besides showing very conclusively that living beings have no greater enemies in nature than the results of the decay of their own bodies. The "pus" in the pleural cavity was, in its chemical state, a sort of nitro-glycerine; and in its situation material foreign to the body of the patient, storing up "force," which seriously threatened the patient's life several times, necessitating its removal by "paracentesis," when "nutrition," *i. e.*, the normal movement of new material into living tissue, was resumed. But the sequel makes known that structural forms had been lost—a terrible word and fate for a living being, old or young—necessitating the use of the drainage tube permanently.

The proceedings of the Central Illinois Medical Society, interesting as they undoubtedly are, as reported, do not offer anything for remark, except that with a clearer understanding of the real pathology—that is, state of the structures—no medical man will remark that "uræmic poison is not a cause" of puerperal convulsions; for the so-called uræmia is actual demonstration of the changes of structure which have already occurred.

Editors' Book Table notices several new books, and appends a list of pamphlets and journals received.

Editorially, the Philadelphia University of Medicine gets another ventilation. The Chicago Board of Health gets aired in no enviable light; and some space is occupied by considerations growing out of a bill before the State legislature to create an "Asylum for Imbeciles." And the suspicion of the editor that there is a "nigger in the woodpile," is, without doubt, correct. Illinois is not the only State, nor Chicago the only city, which groans under the weight of State and municipal "Boards" for various purposes, but generally operated in the interest of some ring or clique, with an amazing amount of cutlery which needs grinding.

The City Mortuary Report closes the number.

It requires some such an analysis as this to bring out the merits and defects of any particular issue of any periodical. And truth compels the admission that, in the present case, it is no mean showing. It is, in truth, a really good number, not wholly without blemishes in the make up and matter of some of its contents. It affords some evidence, at least, that progress is being made in removing the obstructions which so seriously impede the arrival of the "good time coming," when medicine shall be honored of all men. And to hasten which this review has been written, not in the spirit of fault-finding, but with cordial good will to editors and contributors.

Messrs. W. B. Keen, Cooke & Co., the publishers, present the JOURNAL to its readers in faultless typography, on beautiful paper, the whole mechanical execution being an honor to the publishers' art.

